# Mapping the research landscape of nanoparticles and their use in denture base resins: a bibliometric analysis

**DOI:** 10.1186/s11671-024-04037-1

**Published:** 2024-05-30

**Authors:** Ravinder S. Saini, Shashit Shetty Bavabeedu, Syed Altafuddin Quadri, Vishwanath Gurumurthy, Masroor Ahmed Kanji, Abdulmajeed Okshah, Rayan Ibrahim H. Binduhayyim, Mario Alberto Alarcón-Sánchez, Seyed Ali Mosaddad, Artak Heboyan

**Affiliations:** 1 Department of Dental Technology, COAMS, King Khalid University, Abha, Saudi Arabia; 2 Department of Restorative Dental Sciences, College of Dentistry, King Khalid University, Abha, Saudi Arabia; 3 Faculty of Chemical-Biological Sciences, Autonomous University of Guerrero, Chilpancingo de los Bravo, Guerrero Mexico; 4 Department of Research Analytics, Saveetha Dental College and Hospitals, Saveetha Institute of Medical and Technical Sciences, Saveetha University, Chennai, India; 5 Student Research Committee, School of Dentistry, Shiraz University of Medical Sciences, Qasr-e-Dasht Street, Shiraz, Iran; 6 Department of Prosthodontics, Faculty of Stomatology, Yerevan State Medical University after Mkhitar Heratsi, Str. Koryun 2, 0025 Yerevan, Armenia

**Keywords:** Nanoparticles, Denture base resins, Research landscape, Bibliometric analysis, Effect

## Abstract

**Background:**

Nanoparticles are increasingly used in dentistry for various applications, including enhancing the mechanical properties of denture base resins. This study aimed to comprehensively review and analyze the research landscape of nanoparticles and their effect on the flexural strength of denture base resins to identify key research areas and trends and to highlight the importance of collaboration between authors and institutions.

**Methods:**

A Bibliometric Analysis was conducted using the Keywords “Nanoparticle*” AND “Denture*” OR “CAD/CAM.” The literature search from the WOS database was restricted to the publication years 2011 to 2022.

**Results:**

Key findings encompass an increase in research publications but a decline in citations. Saudi Arabia, China, and Iraq led this research, with specific institutions excelling. Notable journals with high impact factors were identified. Authorship patterns show variations in citation impact. Additionally, keyword analysis revealed that current research trends offer insights into influential authors and their networks.

**Conclusions:**

The analysis of nanoparticles and denture base resins reveals a dynamic and evolving landscape that emphasizes the importance of collaboration, staying current with research trends, and conducting high-quality research in this ever-evolving domain.

## Introduction

In dentistry, nanoparticles are increasingly employed to enhance the mechanical properties of resins used in dentures. It is vital to learn about the present state of research in the rapidly developing discipline of dentistry as it moves closer to more advanced technology [1]. Dental prostheses, including partial and complete dentures, are frequently fabricated using denture base resins. However, some challenges associated with denture base resins are their susceptibility to fracture under stress and the need for improved biocompatibility. To address these difficulties, it has been proposed that adding nanoparticles to these materials enhances their mechanical properties and biocompatibility [2, 3].

Nanoparticles are measured in nanometers, typically ranging in size from 1 to 100 nm. These particles can be synthesized from various materials, including metals, polymers, and ceramics, and they exhibit unique physicochemical properties. The integration of nanoparticles in dentistry has been extensively investigated, with numerous studies demonstrating a significant improvement in the mechanical, thermal, and biological properties of dental materials, including denture-base resins [4]. Applications in prosthodontics, conservative dentistry, endodontics, restorative dentistry, orthodontics, dentofacial orthopedics, oral medicine and radiology, preventive dentistry, dental implants, and dentin hypersensitivity have been discussed in the literature [5]. Because of their facile penetration due to their small size, they are useful in dentistry, medical, and medication administration because of their antibacterial, anti-inflammatory, and antioxidant qualities. Research has evaluated their cytotoxicity, concentration effects, and physical characteristics, emphasizing their importance in orthodontics [6].

Their special qualities, like increased surface area and reactivity, make them indispensable for use in prosthetic materials. Conventional materials, such as ceramics and polymers, can be strengthened with nanoparticles to increase their wear resistance and mechanical strength. They also provide targeted medicine delivery, which lowers inflammation and encourages tissue regeneration surrounding the prosthetic implants. In dentistry, the biocompatibility and antibacterial qualities of denture base resins are improved by the incorporation of nanoparticles, which guarantees enhanced oral health for prosthesis users. Nanoparticles are essential for the advancement of prosthetic technology because they provide improved biocompatibility, functionality, and durability [7].

Bibliometric analysis provides a powerful and comprehensive approach to studying the growth and development of a particular research field, tracing the publication history, key research topics, and collaboration patterns among researchers. The bibliometric analysis method utilizes data mining, co-citation analysis, and bibliographic coupling to extract, analyze, and visualize relevant information from scholarly publications. Bibliometric analysis is useful in presenting current and future trends, summarizing findings, and providing insights into research gaps, thereby offering guidance for future research [8, 9].

The primary objective of the present study was to critically analyze the body of literature on nanoparticles in denture base resins, elucidate publication trends, determine global research dynamics, quantify journal impact factor and author citation index, discover emerging themes and content trends, perform collaboration network analysis, and identify bursts of citations.

## Methods

Scientific information was examined through bibliometric methodologies facilitated by Clarivate's Web of Science Database on June 4, 2023. This search comprised research topics, which included titles, abstracts, and keywords. The keywords selected for this study were “Nanoparticle*” AND “Denture*” OR “CAD/CAM;” the search resulted from the WOS database. This search was restricted to the publication years from 2011 to 2022 and was abandoned from any other item in terms of study design.

There were probably a number of considerations when choosing to conduct this study's article search using the Web of Science (WOS) platform. First, the WOS is a well-known and extensive database that spans a wide range of scientific subjects, such as materials science, dentistry, and nanotechnology, making it appropriate for conducting bibliometric analyses in these areas. It provides the most robust indexing for higher-quality peer-reviewed journals of the most diverse subject matter. Furthermore, the WOS makes a vast array of excellent peer-reviewed publications accessible, guaranteeing the validity and dependability of the information obtained. In addition, WOS has sophisticated search capabilities and indexing tools that allow academics to focus their searches on particular standards such as keywords, document type, and publication year. This makes it possible for researchers to precisely target search queries and turn up pertinent literature in their field of interest. Additionally, WOS provides researchers with access to citation data, a crucial component of bibliometric analyses, which they may use to study author-institution collaboration networks, spot significant publications, and look at citation trends.

This study used bibliometric analysis to examine the relationship between denture base resins and nanoparticles. The acquired data were analyzed using the following criteria: publication year, journal, author country or region, author institute, author citation, and keywords. A network map was used to examine the collaboration.

National/regional, institutional, and author collaboration network mapping was performed using the VOS viewer (Version 1.6.19). The Bibliometrix R-package was utilized in RStudio (R version 4.3.1) to create a cooperation network of literature on nanoparticles and denture base resins and a thematic progression map of author keywords and correlations between three factors: keywords, authors, and sources. The references' strongest citation bursts were examined using CiteSpace (6.3.R1).

### Search strategy

**Fig. 1:**
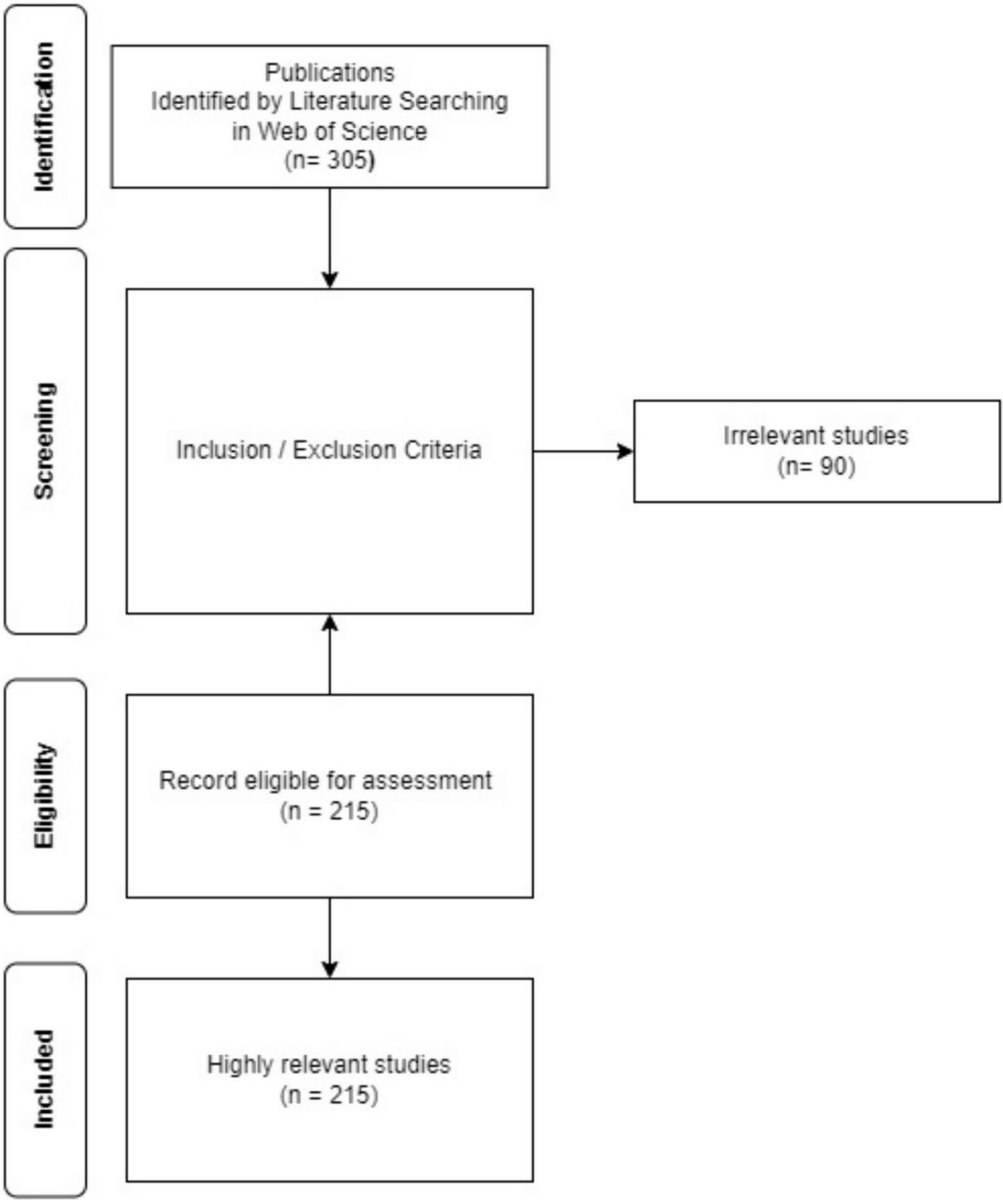
Diagram of four-phase flow

In this study, the search results were filtered based on the publication year, excluding the year 2023. The document types were limited to articles, and the selected languages were restricted to English. This research focused on identifying publications or citations from either the Science Citation Index Expanded (SCI-Expanded) or the Emerging Sources Citation Index (ESCI). This study resulted in 305 publications, and after complete scrutiny of the articles exported from the Web of Science, 215 documents were selected for the final research study (Fig. 1). Ninety documents were not relevant to the papers excluded from this study. Full records (including publication years, document types, authors, affiliations, publication titles, countries/regions, and publishers) and cited references were exported to a tab-delimited text file. For the VOS Viewer, the RIS format for R studio Biblioshiny and Microsoft Excel was used to collect and analyze the data exported from the Web of Science database.

### Inclusion and exclusion criteria

Peer-reviewed scientific literature articles on the impact of nanoparticles on denture base resins were included in this study. The inclusion criteria for certain topics were formulated to ensure their pertinence to the study's focal point of nanoparticles and denture base resins. Articles that had nothing to do with denture base resins or nanoparticles were not subjected to peer review or were not published in English-language scientific journals were disqualified. This exclusion was justified by a number of factors: irrelevant articles would not advance the goals of the study; non-peer-reviewed articles might not have undergone the thorough assessment necessary for scientific validity; and articles not published in English-language scientific journals might not have met standards expected by the scientific community or might present a language barrier.

### Data analysis

215 documents were selected for the final study [10–224]. VOS viewer (Version 1.6.19) analyzed trends for top countries, organizations, and author keywords with the figures. The Bibliometrix R-package in RStudio (R version 4.3.1) software enables the analysis of thematic evolution and examines relationships among three elements (Keywords, Authors, and Sources) and the collaboration network of literature. CiteSpace (6.3.R1) predicts the strongest citation bursts for the publication years. In addition, Clarivate’s Web of Science shows highly cited documents from text files that were exported and predicted in the tables. To conduct comprehensive analyses of the documents obtained from the Web of Science, we thoroughly examined the abstracts and full texts of each paper. This rigorous scrutiny allowed us to identify and retain relevant documents for our research, excluding those that lacked abstracts or authors or were otherwise deemed irrelevant. This allowed us to run the analysis using the bibliometric software tool and have an enrichment context. Necessary clean-up has been done for the keywords to avoid duplicates, for example, the keywords listed by the authors for the documents like nanoparticle or nanoparticles, and this study keeps the plural format as “Nanoparticles.” Extensive scrutiny was conducted to achieve unified and precise facts and analysis. Similar to other studies, a few abbreviations are used in this article for data analysis in various columns of the tables, such as TP stands for total publications, TC for total citations, TLS Total Link Strength, CY citations per year, IF (journal impact factor), and Q quartile category.

## Results

### Examination of the general expansion pattern

**Fig. 2:**
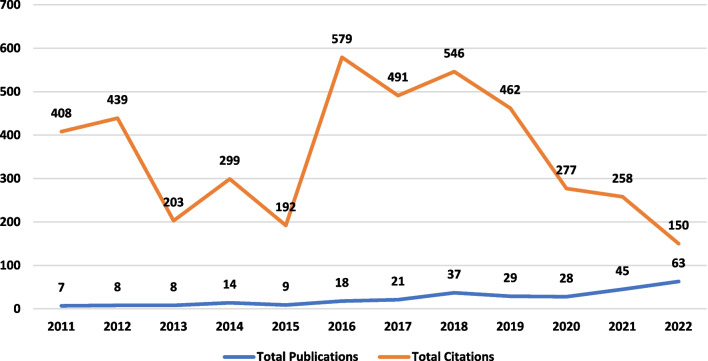
Publishing and citation trends in nanoparticles and denture base resins

Figure 2 shows the general expansion pattern in this dataset, spanning from 2011 to 2022.

### Top countries and organisation for trends in nanoparticles and denture base resins

Table 1 shows the top countries and organizations in terms of trends in the use of nanoparticles in denture base resins. Top countries for trends in nanoparticles and denture base resins using VOS Viewer with criteria of the minimum number of documents of a country (four), the minimum number of citations of a country (four), and out of the 42 countries, 20 met the threshold. Saudi Arabia had the highest ranking, with 38 publications and 542 citations, indicating a significant contribution to research in the specified areas.

**Table 1:** Top countries and organizations on trends in nanoparticles and denture base resins

Rank	Country	TP	TC	TLS	Institution	TP	TC	TLS
1	*Saudi Arabia*	38	542	29	*Imam Abdulrahman Bin Faisal Univ*	22	281	5
2	*Iraq*	23	49	8	*Manchester Metropolitan Univ*	12	132	24
3	*Peoples R China*	21	436	2	*King Saud Univ*	11	205	15
4	*Egypt*	20	374	17	*Univ Manchester*	10	127	23
5	*Romania*	17	282	7	*Sebha Univ*	9	74	21
6	*Turkey*	17	255	4	*Carol Davila Univ Med & Pharm*	8	185	9
7	*Brazil*	14	344	1	*Univ Politehn Bucuresti*	8	185	9
8	*Libya*	14	125	21	*Al Azhar Univ*	7	147	6
9	*England*	13	151	20	*Gazi Univ*	7	71	4
10	*India*	13	74	3	*Univ Baghdad*	7	14	0
11	*Iran*	13	130	2	*Med Univ Warsaw*	6	155	6
12	*Japan*	11	122	8	*Polish Acad Sci*	6	155	6
13	*Poland*	10	283	1	*Univ Belgrade*	6	51	4
14	*Serbia*	10	97	7	*Mersin Univ*	5	66	4
15	*USA*	9	129	11	*Sao Paulo State Univ*	5	130	0
16	*South Korea*	8	233	1	*Babes Bolyai Univ*	4	77	4
17	*Malaysia*	5	51	5	*Coll Med Technol*	4	48	0
18	*Slovenia*	5	23	8	*Iuliu Hatieganu Univ Med & Pharm*	4	77	4
19	*Thailand*	5	55	3	*Keimyung Univ*	4	144	0
20	*Germany*	4	80	3	*Natl Res Ctr*	4	135	2

*TP*, total publication, *TC*, total citation and *TLS*, total link strength

### Journals with significant impact

Table 2 lists the top journals on nanoparticles and denture base resins. Journals with Significant Impact were identified using VOS viewers with the criteria of the minimum number of documents of a source (five) and the minimum number of citations of a source (five); out of the 104 countries, 10 meet the threshold.

**Table 2:** Top journals in nanoparticles and denture base resins

Source	Country	TP	TC	TLS	Publisher	IF	Q
*Polymers*	Switzerland	12	69	29	*MDPI*	4.967	Q1
*Materials*	Switzerland	11	219	41	*MDPI*	3.748	Q1
*Dental Materials Journal*	Japan	9	131	34	*Japanese Soc Dental Materials Devices*	2.418	Q3
*Journal Of Prosthodontics-Implant Esthetic and Reconstructive Dentistry*	USA	9	232	42	*Wiley*	3.485	Q2
*Nanomaterials*	Switzerland	9	143	43	*MDPI*	5.719	Q1
*International Journal of Nanomedicine*	New Zealand	8	300	48	*Dove Medical Press Ltd*	7.033	Q1
*Journal Of the Mechanical Behavior of Biomedical Materials*	Netherlands	6	112	11	*Elsevier*	4.042	Q2
*Materiale Plastice*	Romania	6	36	12	*Revista Chimie SRL*	0.782	Q4
*Revista De Chimie*	Romania	6	49	13	*Chiminform Data S A*	-	-
*Journal Of Prosthetic Dentistry*	USA	5	98	17	*Mosby-Elsevier*	4.148	Q1

*TP*, total publication, *TC*, total citation, *TLS*, total link strength, *IF*, impactfactor and *Q*, journal category

### Authorship pattern

**Fig. 3:**
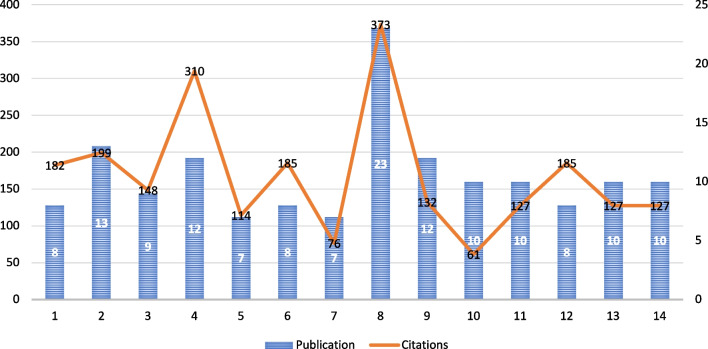
Authorship pattern

Figure 3 shows the authorship pattern using VOS viewers with the minimum number of documents of an author (seven) and the minimum number of citations of an author (seven). Of the 872 authors, 14 met the threshold.

### Authors’ keyword analyses on nanoparticles and denture base resins

**Fig. 4:**
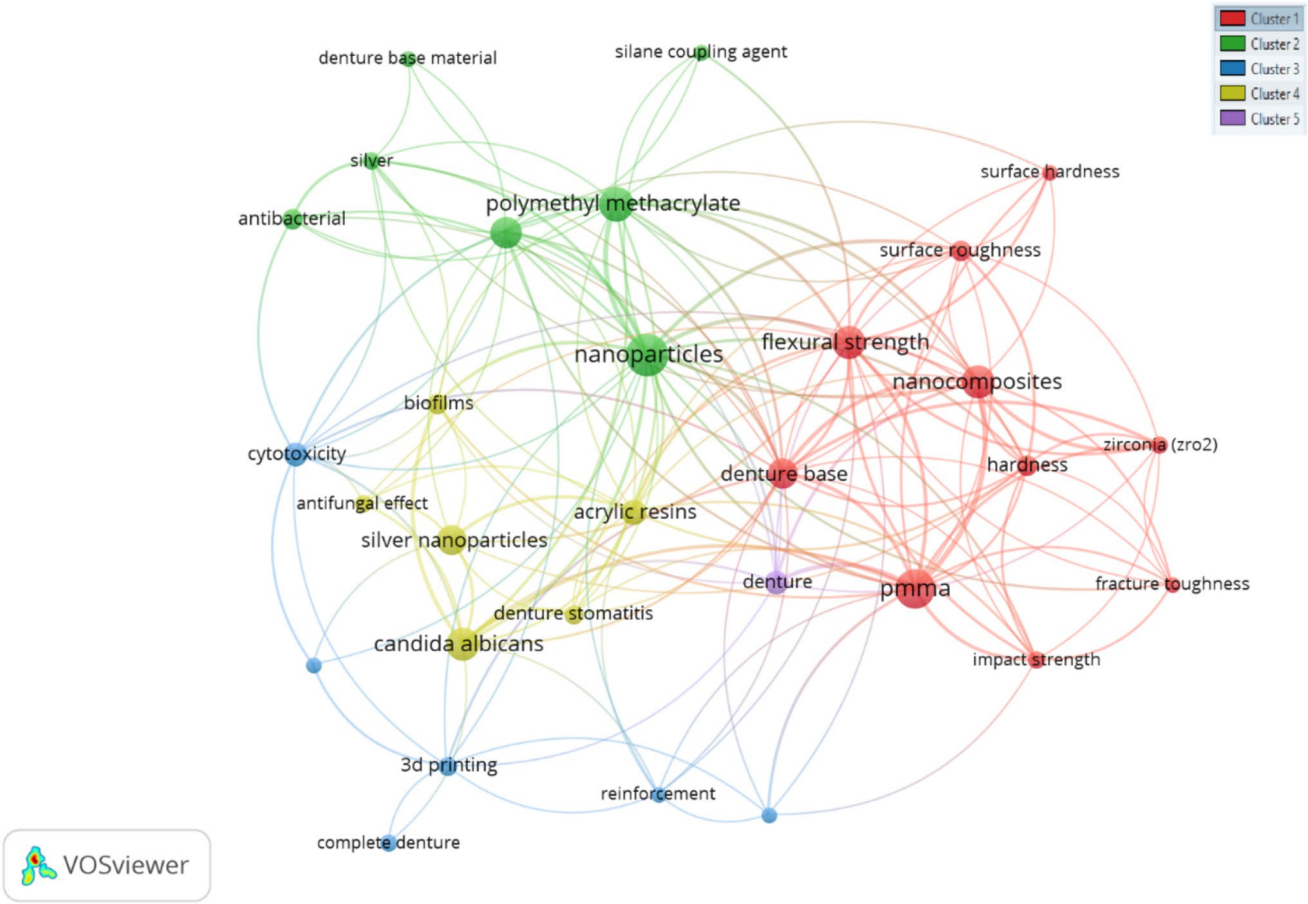
Author keyword relationship network

Figure 4 shows the author’s keyword analyses using VOS viewers with criteria of minimum number occurrences of keywords five. Of the 506 keywords, 30 met the threshold. Five clusters were organized based on connection strength and occurrence, and each color denoted a different cluster.

### Thematic evolution map of author keywords

**Fig. 5:**
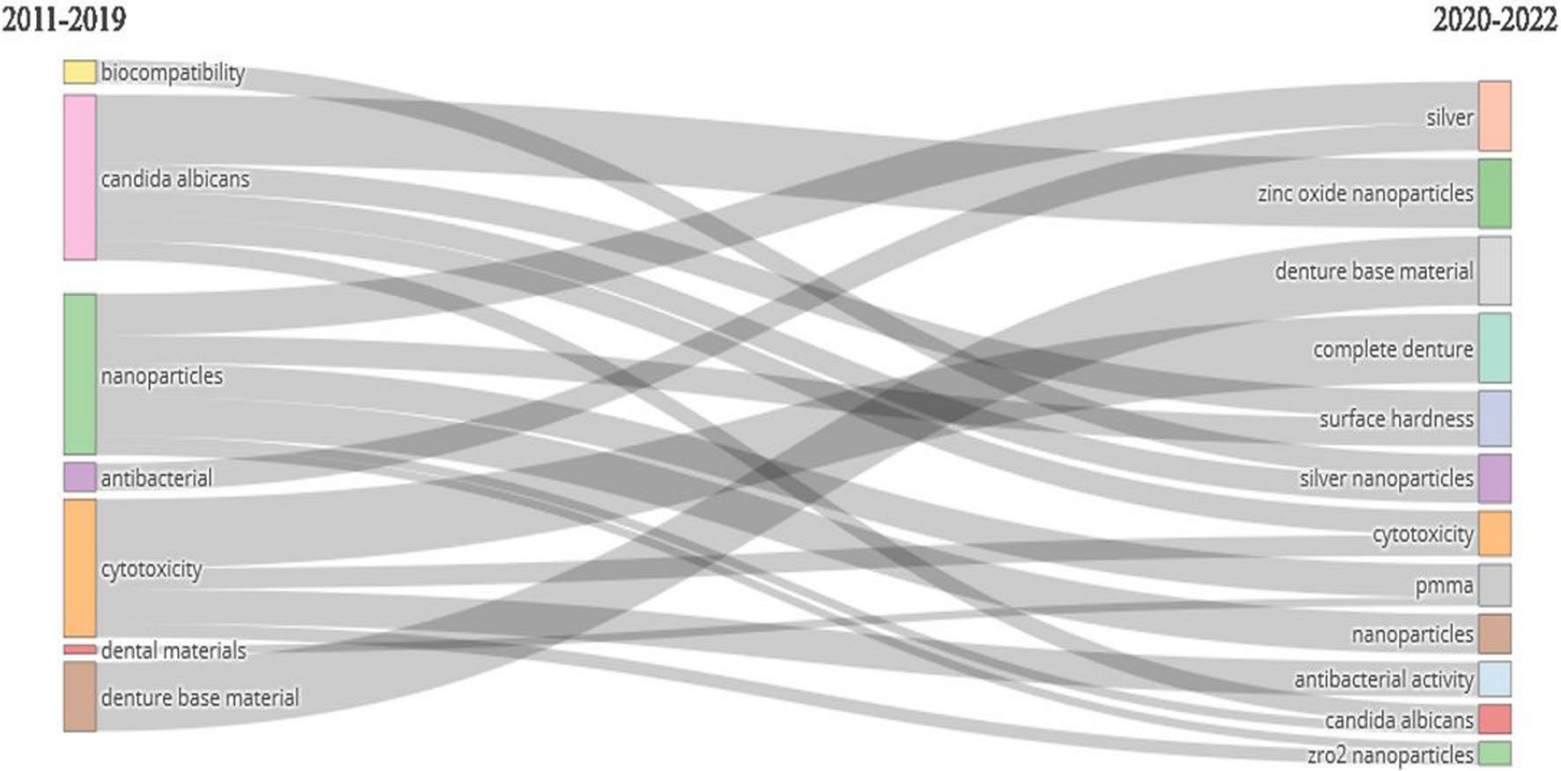
Thematic evolution map of author keywords

The thematic evolution of five keywords over the last 12 years (Fig. 5) showed a clear shift in the nanoparticles and denture base resins. Biocompatibility, antibacterial, and dental materials disappeared by 2022. The results show that silver, zinc oxide nanoparticles, complete dentures, surface hardness, silver nanoparticles, PMMA, and ZrO_2_ nanoparticles are hot topics for 2022. Cytotoxicity, *Candida albicans*, nanoparticles, and denture base material were important keywords throughout the ten years (2011–2022).

### Highly cited articles on nanoparticles and denture base resins

Table 3 shows the top 10 highly cited articles on nanotechnology and denture base resins minimum using VOS Viewers with criteria number of citations of a document 10. Of the 2017 documents, 92 met this threshold.

**Table 3:** Top 10 highly cited articles on nanotechnology and denture base resins

Title	Authors	Source	TC	TLS	Year
*Silver Distribution and Release from an Antimicrobial Denture Base Resin Containing Silver Colloidal Nanoparticles*	Monteiro et al	Journal Of Prosthodontics-Implant Esthetic and Reconstructive Dentistry	96	4	2012
*Cytocompatible Antifungal Acrylic Resin Containing Silver Nanoparticles for Dentures*	Acosta-Torres et al	International Journal of Nanomedicine	95	16	2012
*Poly (Methyl Methacrylate) With TiO*_*2*_* Nanoparticles Inclusion for Stereolithographic Complete Denture Manufacturing—The Future In Dental Care For Elderly Edentulous Patients?*	Totu et al	Journal Of Dentistry	89	13	2017
*Evaluation Of Candida Albicans Adhesion and Biofilm Formation on A Denture Base Acrylic Resin Containing Silver Nanoparticles*	Wady et al	Journal Of Applied Microbiology	86	9	2012
*Antifungal Activity of Denture Soft Lining Material Modified by Silver Nanoparticles-A Pilot Study*	Chladek et al	International Journal of Molecular Sciences	71	7	2011
*Reinforcement of denture base PMMA with ZrO*_*2*_* nanotubes*	Yu et al	Journal Of the Mechanical Behaviour of Biomedical Materials	64	10	2014
*Influence Of Incorporation of ZrO*_*2*_* Nanoparticles on The Repair Strength of Polymethyl Methacrylate Denture Bases*	Gad et al	International Journal of Nanomedicine	62	11	2016
*Effect Of Zirconium Oxide Nanoparticles Addition on The Optical and Tensile Properties of Polymethyl Methacrylate Denture Base Material*	Gad et al	International Journal of Nanomedicine	62	13	2018
In Vitro* Antimicrobial Effect of The Tissue Conditioner Containing Silver Nanoparticles*	Nam	Journal of Advanced Prosthodontics	61	2	2011
*Effect Of a Denture Base Acrylic Resin Containing Silver Nanoparticles on Candida Albicans Adhesion and Biofilm Formation*	Li et al	Gerodontology	54	9	2014

*TC*, total citation and *TLS*, total link strength

### Relationships between three factors (keywords, authors, and sources)

**Fig. 6:**
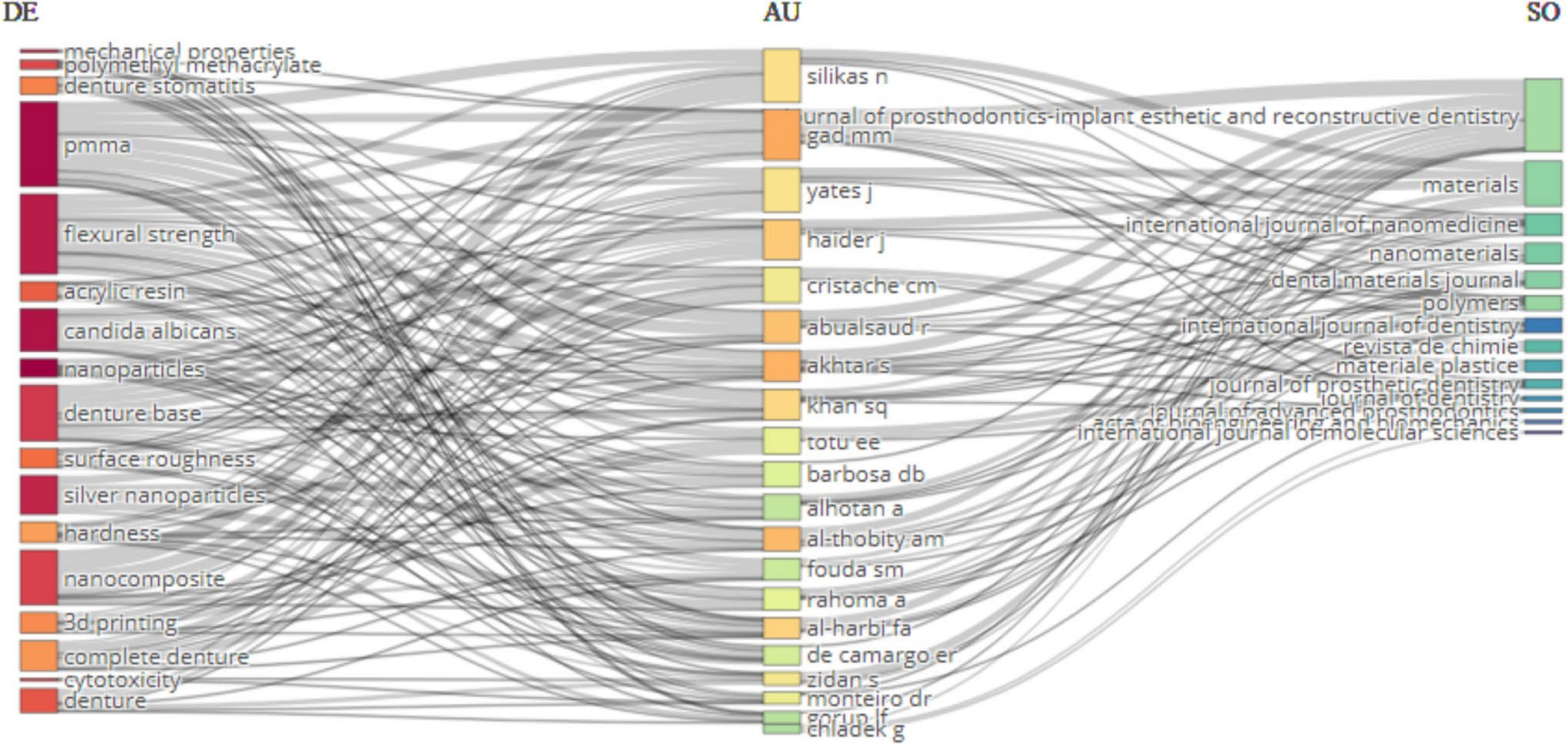
Relationships between three factors (Keywords, Authors, and Sources)

A comprehensive three-factor analysis was conducted to investigate the intricate interplay between sources (on the right), authors (in the middle), and keywords (on the left), shedding light on the publication preferences and collaborative patterns within the field, as shown in Fig. 6. Notably, the journals "Journal of Prosthodontics-Implant Esthetic and Reconstructive Dentistry," "Materials," and "International Journal of Nanomedicine" exhibited strong and consistent collaborative ties with three specific authors: Gad MM, Yates, Haider J, and Silikas N.

### Collaboration network of literature on nanoparticles and denture base resins

**Fig. 7:**
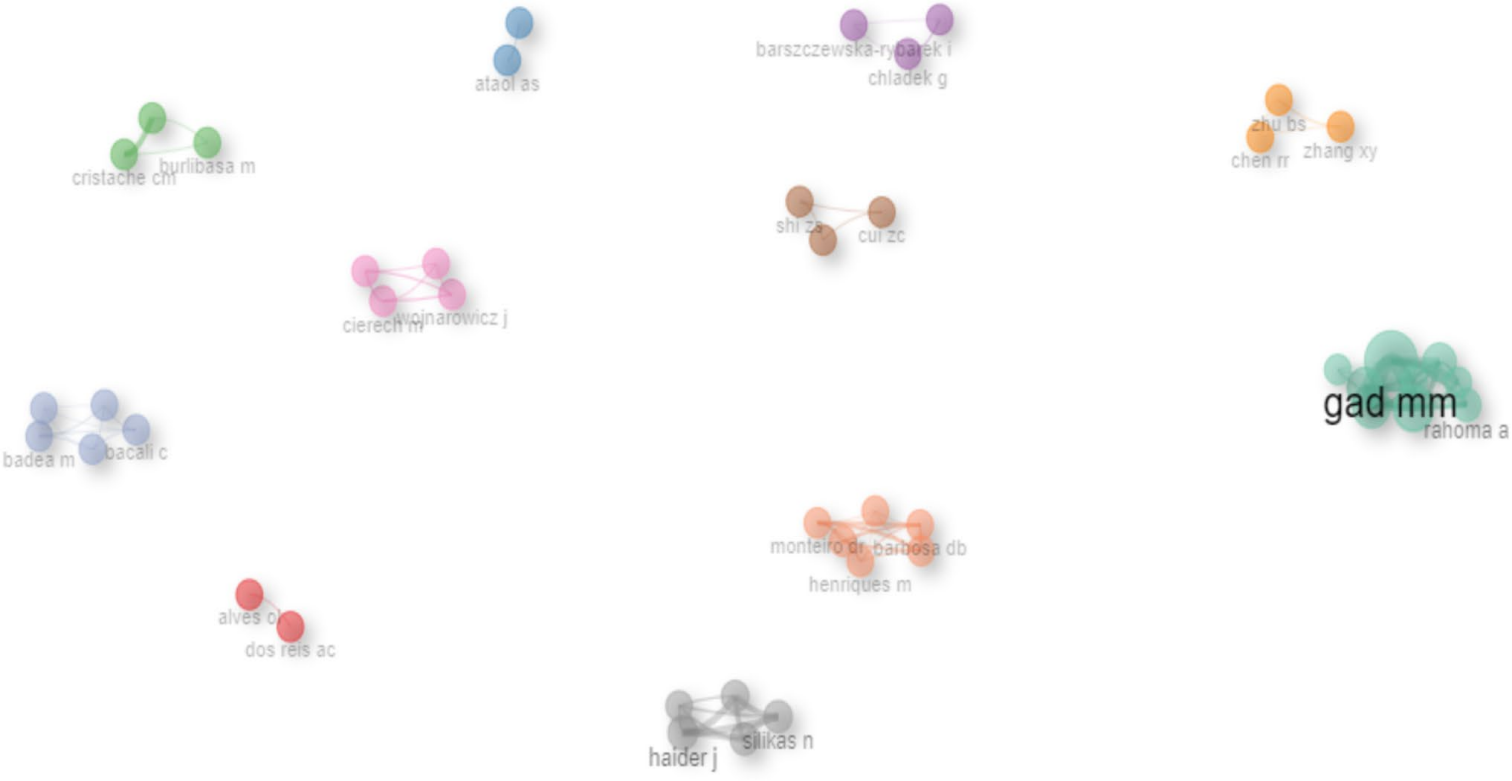
Collaboration network of literature on nanoparticles and denture base resins

Figure 7 shows the collaboration network within the literature on nanoparticles and denture base resins, specifically focusing on nanoparticles and denture base resins, revealing valuable insights into the relationships and collaborative efforts among authors in this field.

#### Citation bursts of literature on nanoparticles and denture base resins

**Fig. 8:**
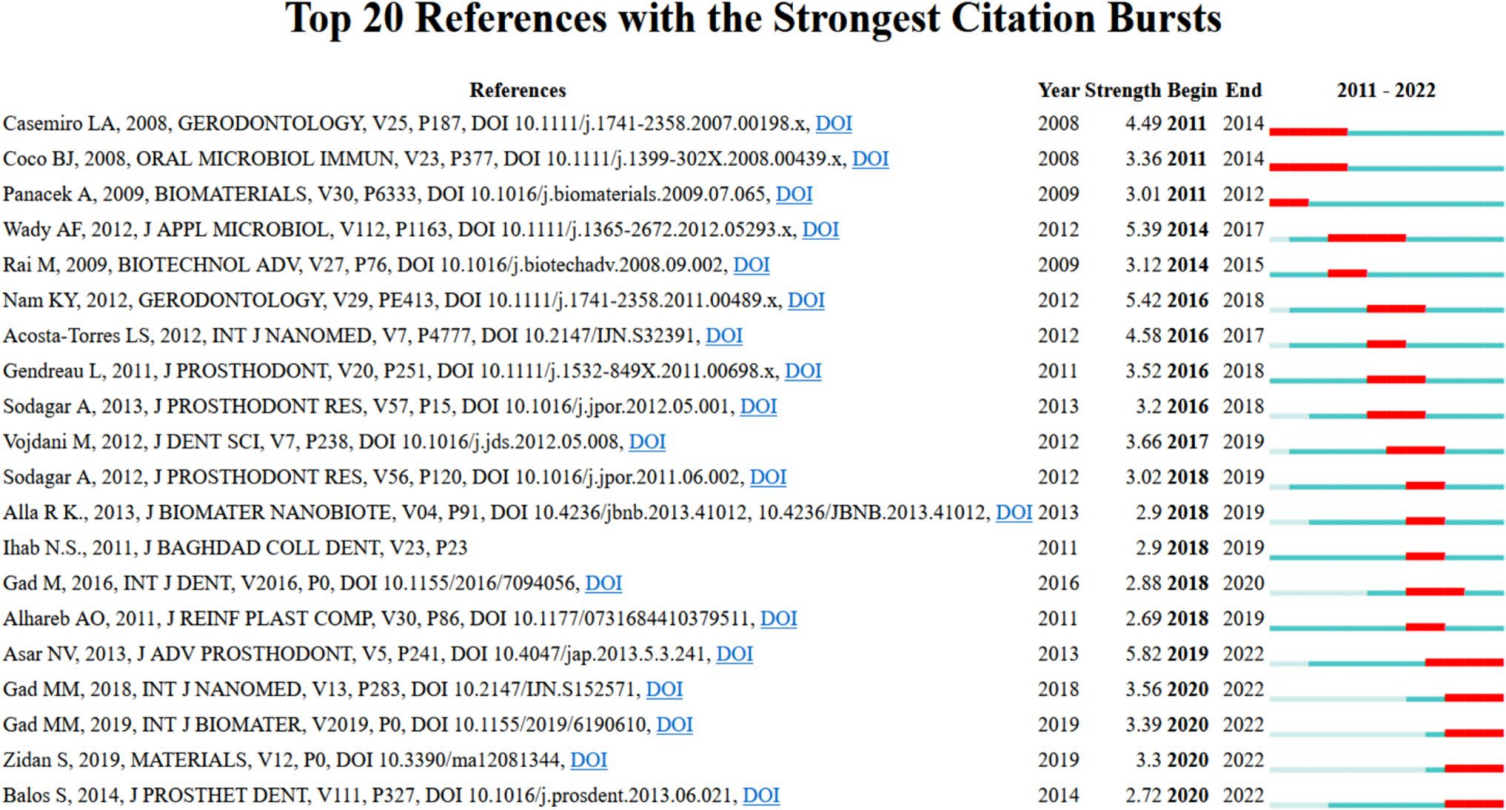
Citation Bursts of Nanoparticles and Denture Base Resins (2011–2022)

Figure 8 shows that references on nanoparticles and denture base resins, spanning from 2008 to 2019, experienced varying levels of citation bursts between 2011 and 2022. Acosta-Tormi LS's 2012 publication garnered substantial attention with a burst strength of 5.42 from 2016 to 2018, followed closely by A NV's 2013 paper with a burst strength of 5.82 between 2019 and 2022.

## Discussion

### Examination of the general expansion pattern

The dataset analysis (Fig. 2) covering 2011 to 2022 reveals a consistent rise in publications from 7 in 2011 to 63 in 2022, signifying a substantial increase in research output. Nevertheless, a paradoxical decline in total citations is observed, peaking at 579 citations in 2016 and decreasing to 150 citations in 2022. Several factors, such as potential field saturation, changes in citation concentration, variations in database coverage, topic diversification, global citation inequalities, and declining citation accuracy, may explain the divergence between publication growth and declining citation impact. Other studies have shown that publications in rapidly expanding fields typically receive more citations than those in slowly expanding fields. Future citation counts may be affected by the growth rate of topics, indicating that the impact of citations may be influenced by the nature of the study topic itself [225]. There is a general tendency in research towards greater collaboration, which corresponds to the rise in citation concentration. The distribution of citations among publications may be impacted by collaboration, which can have an impact on citation patterns [226]. Researchers should consider these complexities when assessing the significance of their work and recognize that citation counts alone may not adequately reflect the true impact of their publications in the broader research landscape. This data may indicate the need for further investigation into the quality and relevance of the research conducted in this field.

### Top countries and organisation for trends in nanoparticles and denture base resins

The analysis of top countries and organizations in terms of nanoparticles, denture base resins, and flexural strength revealed interesting trends and patterns (Table 1). In terms of countries, Saudi Arabia has emerged as a standout contributor, leading in the number of publications (38) and citations (542), significantly impacting research in these domains. The People's Republic of China and Iraq follow closely, with 21 and 23 publications, respectively. China has many publications but relatively fewer citations, whereas Iraq has fewer publications but a higher citation count. The unexpected positioning of the United States in 15th place with nine publications and 129 citations prompts further investigation to understand the contextual factors influencing this ranking. Iran and Japan are also noteworthy, with 13 and 11 publications, respectively. Germany is in the 20th position with four publications and 80 citations. According to an additional study, the United States, the Netherlands, and the United Kingdom are the top three nations in the world for denture base resins and nanoparticle research. To improve the mechanical qualities and general performance of denture base resins, these nations have been actively involved in studies concentrating on the addition of nanoparticles, such as silicon dioxide, titanium dioxide, and zirconium dioxide, to the resin [227, 228].

The top organization for trends in nanoparticles, denture base resins, and flexural strength, using VOS Viewer with criteria of a minimum number of documents of an organization (four), a minimum number of citations of an organization (ten) and out of the 280 countries, 23 met the threshold. The provided data outline the research performance of several academic institutions on nanoparticles, denture base resins, and flexural strength. Imam Abdulrahman Bin Faisal University took the lead, with 22 publications and 281 citations, indicating a robust presence in research on nanoparticles, denture base resins, and flexural strength. Manchester Metropolitan University followed closely, with 12 publications and 132 citations, boasting the highest TLS at 24, suggesting a notable overall impact in these specialized fields. King Saud University and the University of Manchester also exhibited strong research output, with 11 and 10 publications, respectively. The diversity in the range of institutions involved suggests collaborative and global efforts to advance research in these domains. Additional study reveals Reputable academic institutions that have published studies on nanoparticles and denture base resins include the Brazilian universities of São Paulo and Campinas. Research on the effects of nanoparticles on the mechanical characteristics of denture repairs and the creation of denture base materials utilizing nanofillers in 3D-printed dentures have been intensively pursued by these colleges [229]. These results provide valuable insights for researchers and policymakers, enabling a better understanding of global distribution, impact, and collaboration patterns in specific research areas. Variations in country and organization rankings prompt further exploration of the dynamics influencing these trends, facilitating more targeted and informed research efforts in the future.

### Journals with significant impact

The analysis of journals with significant impact provides valuable insights into the prominence of publications on polymers, materials, dental materials, prosthodontics, implant esthetics, reconstructive dentistry, nanomaterials, and nanomedicine (Table 2). Among the ten countries meeting the established threshold, MDPI's "Polymers" and "Materials," originating from Switzerland, emerged as influential contributors. These journals exhibited substantial Total Publications (TP), Total Citations (TC), and Total Link Strength (TLS), indicating a robust overall impact on research in the specified domains. The competitive Impact Factors (IF) in Q1 further underscore their significance. Additionally, MDPI's "Nanomaterials" demonstrate a comparable level of impact, solidifying MDPI's position as a publisher with a notable presence in these research areas. The "International Journal of Nanomedicine" from Dove Medical Press Ltd in New Zealand stands out with an exceptional Impact Factor of 7.033 and a high TC of 300, highlighting its influential role in the nanomedicine field. Noteworthy contributions also come from discipline-specific journals, such as the "Dental Materials Journal" from the Japanese Society of Dental Materials Devices and the "Journal of Prosthodontics-Implant Esthetic and Reconstructive Dentistry" from Wiley in the USA. These journals exhibited substantial TC and TLS values, emphasizing their impact on research in dental materials, prosthodontics, and other related disciplines. However, another study indicates that the National Center for Biotechnology Information is the top journal in the field of published papers on nanoparticles and denture base resins. Studies on the impact of nanoparticles on the mechanical characteristics of acrylic denture repairs and the effects of adding nanoparticles to dental acrylic resins on their antibacterial and physicomechanical properties have been conducted in large part on the NCBI [230]. Such studies have made a substantial contribution to our knowledge of how nanoparticles might improve the dimensional stability, strength, color, and antibacterial qualities of denture base resins, opening the door for new developments in denture production and repair [231]. These results suggest a concentration of impactful research in journals affiliated with specific publishers and countries. This information is valuable for researchers and practitioners seeking to stay abreast of influential publications in these specialized domains, facilitating a deeper understanding of the current state and trends in the respective fields. The diversity of influential journals highlights the collaborative and interdisciplinary nature of research in polymers, materials, dentistry, and nanotechnology.

### Authorship pattern

The findings from the authorship pattern offer valuable insights into the research landscape of nanoparticles, denture base resins, and flexural strength (Fig. 3). Among the 872 authors analyzed, 14 met the predefined threshold, signaling their notable impact in the field. The observed multiple instances of publications by specific authors, such as those associated with Publications 8, 12, and 10, highlight a consistent and recurring contribution from these authors across various studies. The varying citation counts accompanying these publications suggest a range of impacts and a recognition of their contributions. Publication 23 stands out with a remarkable citation count of 373, indicating a substantial influence on the scholarly community. Other studies, however, have demonstrated that authorship pattern analysis singled out particular writers who have had a considerable influence on the academic community by contributing significantly to the fields of nanoparticles, denture base resins, and flexural strength research. Notably, authors A.A.A., M.M.G., A.K., I.A.A., and M.H.A. were among those who made substantial contributions to this field of study. The development of 3D-printable nanocomposite denture-based resins is appropriate for clinical use. The impact of ZrO_2_ nanoparticles on the mechanical and surface qualities and 3D-printed nanocomposite denture-based resins was among the subjects of their work. These authors’ creative research and contributions to the fields of prosthodontics and dental materials have been essential in expanding our understanding of denture base materials and improving the quality of dental prostheses [232]. This authorship pattern analysis underscores the significance of certain contributors who have consistently made meaningful contributions to the body of knowledge in these research areas. Identifying these prolific authors and their recurring impacts provides a basis for further exploration of the specific themes and innovations they bring to the field. Moreover, understanding the dynamics of collaboration and the interplay between these influential authors could reveal patterns that contribute to the advancement of research on nanoparticles and denture base resins. These results can guide future research initiatives and collaboration strategies and highlight potential leaders in these specialized domains.

### Authors’ keyword analyses on nanoparticles, denture base resins, and flexural strength

Keywords are crucial terms that play a significant role in nanoparticles, denture base resins, and flexural strength (Fig. 4). Establishing a minimum threshold ensures that only the most prevalent and meaningful keywords are considered, providing a concise and focused representation of the key themes in the literature. The organization of these keywords into five clusters based on connection strength and occurrence suggests a certain level of interconnectedness and commonality among them. The distinct colors assigned to each cluster likely represent different thematic groupings or aspects within the overarching topics. Analyzing the composition and relationships within each cluster can reveal patterns, trends, and potential interdisciplinary intersections in the research landscape. This keyword analysis is valuable for researchers because it highlights the core terminology that defines and characterizes discussions in specific research areas. Understanding the clustering and distribution of these keywords can guide scholars in navigating the literature, identifying prevalent themes, and recognizing the key factors shaping research on nanoparticles, denture base resins, and flexural strength. Additionally, the results offer a basis for further exploration, enabling researchers to delve deeper into specific clusters or keywords for more detailed investigation and analysis. Another study examined the effect of ZrO_2_ nanoparticles on the flexural strength of a conventional, heat-cured PMMA denture base material. The addition of 1 wt. of ZrO_2_ nanoparticles improves the flexural strength of the material [233]. Other studies have examined the impact of nanoparticles on wear resistance and microhardness, two other mechanical qualities of denture base materials, and their effects on flexural strength. The antifungal and antibacterial properties of nanoparticles in denture base materials have also been the subject of several studies. Overall, the use of nanoparticles in denture base materials shows promise in improving their mechanical properties and potentially reducing the occurrence of denture fractures. However, further research is needed to fully understand the effects of different types and concentrations of nanoparticles on denture base materials and to ensure their safety for use in the oral cavity.

### Thematic evolution map of author keywords

The thematic evolution of keywords over the last 12 years showed a clear shift in nanoparticles and denture base resins. Biocompatibility, antibacterial, and dental materials disappeared by 2022 (Fig. 5). The results show that silver, zinc oxide nanoparticles, complete denture, surface hardness, silver nanoparticles, PMMA, and ZrO_2_ nanoparticles are hot topics in 2022. Additionally, cytotoxicity, *Candida albicans*, nanoparticles, and denture base material were important keywords throughout the ten years (2011–2022). This shift in research focus may be due to several factors, including the emergence of new technologies and materials, changing patient needs, and advancements in research methods. For example, the use of nanoparticles in denture base resins has gained attention owing to their potential to improve mechanical and surface properties [232, 234]. The antifungal properties of chitosan have also been studied in denture-based resins [235]. Additional research indicates that there has been a movement in the thematic progression of keywords in denture base material research towards the utilization of nanoparticles, specifically zinc oxide and silver nanoparticles, in denture base resins. This change reflects the increased interest in applying nanoparticles to improve the surface and mechanical characteristics of denture base materials. To increase the functionality and biocompatibility of denture base materials, researchers have focused on cutting-edge substances and techniques, such as 3D-printed nanocomposite denture base resins and bioactive glass-enhanced resins [236].

The overall theme progression of keywords over the past 12 years indicates that denture base resin and nanoparticle research is dynamic and evolving. In addition to examining the biocompatibility and cytotoxicity of these materials, researchers are investigating novel materials and methods to enhance the mechanical and surface qualities of denture base-resins. Research on the application of nanoparticles in denture base resins is intriguing; however, further investigation is required to fully understand the advantages and disadvantages of this approach.

### Highly cited articles on nanoparticles and denture base resins

Analyzing the top 10 highly cited articles on the intersection of nanotechnology and denture base resins provides valuable insights into impactful research in this specialized field (Table 3). Monteiro et al.'s article, "Silver Distribution and Release from an Antimicrobial Denture Base Resin Containing Silver Colloidal Nanoparticles," stands out as the most cited, with 96 citations and a Total Link Strength (TLS) of 4. This indicates their widespread influence and relevance in the scientific community. Acosta-Torres et al.'s work on "Cytocompatible Antifungal Acrylic Resin Containing Silver Nanoparticles for Dentures" (International Journal of Nanomedicine, 2012) closely followed 95 citations and a higher TLS of 16, showing not only citation numbers but also a strong network impact, possibly indicating widespread discussion or collaboration. Another noteworthy article is Totu et al.'s "Poly (Methyl Methacrylate) With TiO_2_ Nanoparticles Inclusion for Stereolithographic Complete Denture Manufacturing" (Journal of Dentistry, 2017), which, with 89 citations and a TLS of 13, has a substantial impact on the field. These highly cited articles, published across diverse journals, collectively underscore the significant interest in and impact of research on the confluence of nanotechnology and denture base resins. While adding nanoparticles to denture base resins has many advantages, there are also some risks and disadvantages. Ingestion or inhalation of nanoparticles, particularly certain varieties, such as graphene oxide, titanium dioxide, and silver, may result in adverse health effects. Exposure to these nanoparticles may cause damage to the kidneys, liver, brain, skin, and lungs, among other organs [237]. The multidisciplinary nature of these studies reflects a collaborative effort to advance the scientific understanding and development of advanced dental materials. This analysis provides a foundation for researchers, practitioners, and policymakers to identify key contributions and trends in this evolving and impactful field, guiding future research and applications in nanodentistry and biomaterials.

### Relationships between three factors (keywords, authors, and sources)

The three-factor analysis delves into the complex dynamics among sources, authors, and keywords, revealing intriguing insights into publication preferences and collaborative patterns within the field of nanoparticles and denture base resins (Fig. 6). Notably, three specific journals, "Journal of Prosthodontics-Implant Esthetic and Reconstructive Dentistry," "Materials," and "International Journal of Nanomedicine," are strongly associated with authors Gad MM, Yates, Haider J, and Silikas N, indicating consistent collaborative ties. These authors also consistently employed keywords such as "PMMA," "flexural strength," "nanocomposite," and "denture" in their research, reflecting their significant expertise and interest in these topics. These findings offer valuable insights into publication trends and collaborative networks within the literature, contributing to a deeper understanding of the research landscape and knowledge dissemination by illuminating the relationships between authors, sources, and keywords. These collaborative research patterns can significantly influence the quality of the research publications. On the positive side, collaboration can facilitate the convergence of diverse perspectives, enabling the generation of innovative and high-quality research outcomes. Moreover, it provides researchers with increased access to essential resources, including funding, equipment, and data, ultimately contributing to high-quality research. Collaborative efforts can also foster the development of enhanced research methodologies, further elevating the quality of the publications.

Despite being a popular keyword in our search, there were not many articles about CAD/CAM denture manufacturing, which raises a few possible reasons. One explanation might be that in comparison to other dental specialties, prosthodontics is a relatively new discipline in which CAD/CAM technology has been used. The adoption of digital workflows in this field may have been hampered by the established nature of traditional denture production methods and the high costs of training and technology. Furthermore, the design of a complete denture and the variation in anatomical features specific to each patient provide special difficulties that are not entirely solved by the currently available CAD/CAM methods. Furthermore, studies on other CAD/CAM dental topics, including fixed prosthodontics, may receive more attention in the literature than they do, which could result in an underrepresentation of research that specifically investigates denture manufacturing. Notwithstanding these difficulties, the realization of the potential advantages of digital workflows in terms of increasing accuracy and productivity emphasizes the significance of upcoming research projects aimed at investigating novel ways to utilize CAD/CAM technology to augment the caliber and clinical results of full-denture prostheses. The use of Computer-Aided Design/Computer-Aided Manufacturing (CAD/CAM) technology in denture manufacturing has significantly evolved over time. Initially introduced in 1989 for the in-office fabrication of ceramic restorations, CAD/CAM technology has revolutionized the field of dentistry. The CAD/CAM technology entered the dental sector in the late 1980s, primarily for fabricating ceramic restorations. Over the past 25 years, there has been a substantial increase in its utilization [238].

However, it is essential to acknowledge potential downsides, as collaborative research can sometimes lead to authorship disputes and the pernicious influence of groupthink, where conformity to group opinions can compromise critical evaluation. Therefore, researchers should be mindful of these dynamics and strive to collaborate to enhance the quality of their research publications.

### Collaboration network of literature on nanoparticles and denture base resins

The nanoparticle and denture base resin literature collaboration network provides valuable insights into the authors' relationships and collaborative efforts in this field (Fig. 7). The analysis revealed that numerous authors established strong collaborative connections, forming 11 distinct clusters within the network. These clusters often represent collaboration within specific research teams, institutions, or geographic regions. Furthermore, the network's influential authors or central nodes underscore their significant contributions and leadership in the nanoparticle and denture base resin domains. Notable contributors such as Gad MM, Ellakany P, Abdulsaud R, Althobity AM, Akhtar S, Rahon, Khan SQ, and Alharbi FA, as well as Müller FA, have played pivotal roles in advancing the field [228]. Furthermore, it has been demonstrated that adding nanoparticles, such as titanium dioxide nanoparticles, to 3D-printable resin improves the biocompatibility, wear resistance, stiffness, and strength of the material. TNPs can function as reinforcing agents, giving printed materials more sturdiness and strength while also decreasing surface roughness and boosting wear resistance [227, 239].

Collaboration network analysis provides a visual representation of the collaborative landscape in the nanoparticle and denture base resin field, which can help researchers, practitioners, and institutions comprehensively understand the field's current state. Identifying key contributors and influential authors can help researchers and institutions identify potential collaborators and leaders in the field. The formation of clusters within a network can help researchers identify research areas that are currently receiving significant attention and collaboration. The analysis is limited to authorship and does not consider other factors that influence cooperation, such as funding sources or institutional affiliations. The analysis is limited to a specific set of authors and may not represent the entire field of nanoparticles and denture base resins. The analysis did not provide information on the quality or impact of the research produced by the authors, or the clusters identified in the network. In summary, the collaborative network analysis of nanoparticles and denture base resins provides valuable insights into author relationships, collaborations, and influential figures. While there are limitations to the analysis, it can still be a useful tool for researchers, practitioners, and institutions looking to gain information.

#### Citation bursts of literature on nanoparticles and denture base resins

References on nanoparticles and denture base resins, spanning from 2008 to 2019, experienced varying levels of citation bursts between 2011 and 2022 (Fig. 8). Notably, Acosta-Tormi LS's 2012 publication garnered substantial attention with a burst strength of 5.42 from 2016 to 2018, followed closely by A NV's 2013 paper with a burst strength of 5.82 between 2019 and 2022. These references seem to have sustained interest in the topic over extended periods. Other references, such as Casemiro LA [240], Coco BJ [241], from 2008, and Panacek A [242], had citation bursts between 2011 and 2014, indicating notable peaks of interest during that period. In contrast, several references, such as Alla [243]. and Ihab N.S. [244], experienced smaller citation bursts, suggesting more modest but still noteworthy surges in interest in their respective years. Moreover, Gad [82] saw moderate citation bursts, albeit with less intensity. Lastly, Balo S [167] and the last reference in the list had moderate citation bursts between 2020 and 2022, showing recent trends in the field. These citation bursts reflect the evolving and dynamic interest in research on nanoparticles and denture base resins over the years. The development of novel materials in this arena has been greatly affected by citation bursts on denture base resins and nanoparticles. Studies that have been instrumental in improving the mechanical characteristics, antimicrobial effects, flexural strength, hardness, translucency, surface roughness, cytotoxicity, fracture toughness, and impact strength of denture base resins have focused on the incorporation of metal nanoparticles, including silver, ZrO_2_, nanoparticles, and fibers [245].

Overall, the citation bursts of references on nanoparticles and denture base resins reflect the evolving and dynamic nature of the research in this field. Researchers should be aware of the trends and patterns in citation bursts and strive to conduct high-quality research to advance knowledge in this field. In summary, the current study on denture-based nanomaterials shows promising results in four main areas. First, a high citation count and bursts of citations indicate noteworthy attention and favorable reception, which point to discoveries that have an impact. Second, the benefits of using nanomaterials are further supported by authorship patterns and collaborative networks, which have regular contributions from scholars and journals. Thirdly, thematic evolution maps draw attention to terms like "biocompatibility" and "antibacterial properties," which show that researchers are still interested in this area and have had success employing nanomaterials to improve denture base features. Based on increased citations, collaborative efforts, and theme trends, these observations collectively point to developments and improvements in denture base-material qualities enabled by nanomaterials.

## Limitations

Despite these findings, it is important to recognize some limitations of this study. The use of the Web of Science database as a search engine might introduce a bias by indexing authors from English-speaking countries and excluding non-English publications. The exclusion of studies published in 2023 and before necessarily misses out on recent trends. The search criteria might inadvertently exclude relevant studies from the list of top studies. VOS Viewer and CiteSpace have limitations in identifying the trends, and the outcomes are not entirely objective. Data extraction procedures might introduce errors that ultimately impact the validity of conclusions. Scholars should interpret findings with caution, considering these limitations, and attempt to address them in future studies to cross-validate the trends by employing multiple databases and searching through diverse scholarly literature.

## Conclusions

This bibliometric study in dental and materials science research demonstrates the necessity of researchers looking further into publications and citation patterns. Global collaboration is a growing phenomenon in research, especially in Saudi Arabia, China, and Brazil, and the study points to the collaborative nature of dental materials science. Researchers should focus on the specific contributions of publications, move beyond impact factors, and follow the latest trends, including nanoparticles and mechanical properties. Analysis of collaborative networks and citation bursts offers opportunities for further research directions. Overall, the study offers valuable implications for researchers to navigate the complexities and opportunities in the field effectively.

## Data Availability

The data that support the findings of this study are not openly available due to sensitivity reasons and are available from the corresponding author upon reasonable request.
